# Supplementary material for: Dual-color emissive OLED with orthogonal polarization modes
Source: Nat Commun. 2024 Feb 13;15:1331. doi: 10.1038/s41467-024-45311-1 (PMC10864411; doi:10.1038/s41467-024-45311-1)
Supplement: Supplementary file 1 — Supplementary Information [file 41467_2024_45311_MOESM1_ESM.pdf]

Supplementary Information

Dual-color emissive OLED with orthogonal polarization modes

Ruixiang Chen[1], Ningning Liang[1]*, Tianrui Zhai[1]*

[1] Faculty of Science, Beijing University of Technology, Beijing 100124, China

*Correspondence to: liangnn2020@bjut.edu.cn (N. N. L.), trzhai@bjut.edu.cn (T. R. Z.)

Supplementary Figures

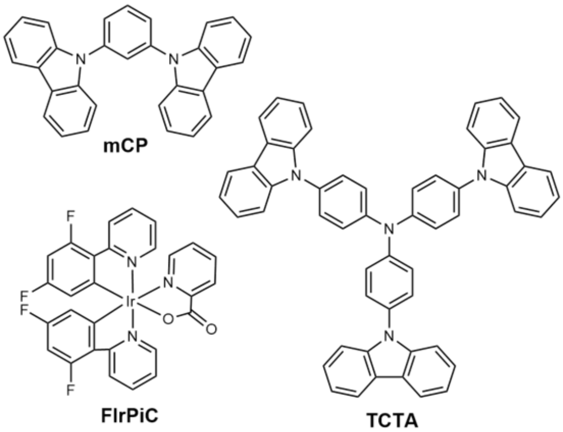

Supplementary Figure 1. Molecular structure of emitting layer materials. The mCP and TCTA are the main materials, while FIrPiC is the guest material.

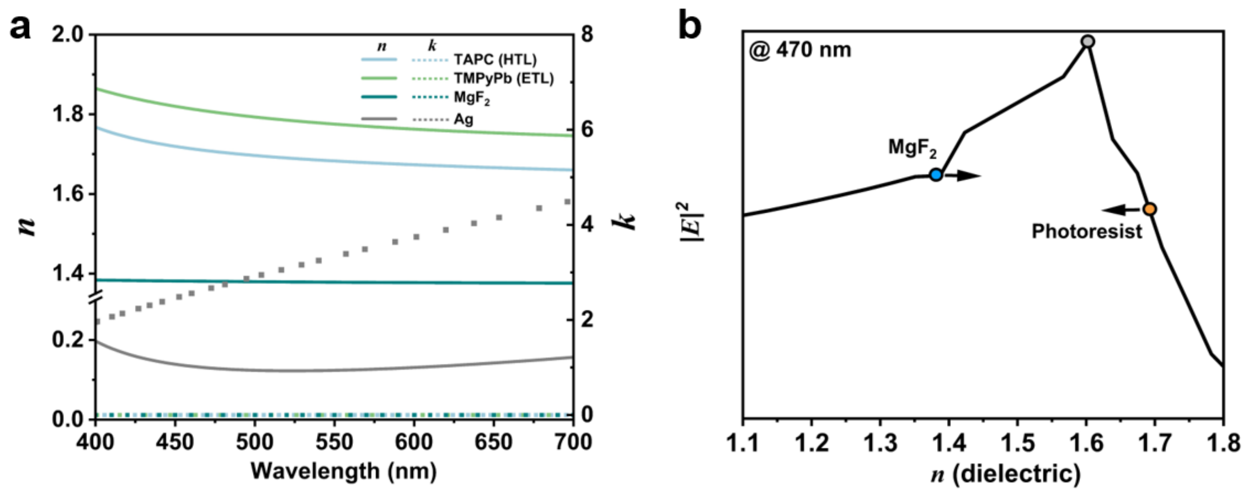

Supplementary Figure 2. Design of Ag/MgF_2 grating. (a) The refractive index (*n*) and extinction coefficient (*k*) of hole transport layer (HTL, light blue), electron transport layer (ETL, green), MgF_2 materials (dark green)and Ag film (grey). (b) The simulated far-field intensity distribution of corrugated OLED with the increasing refractive index of D/M grating. Observation angle is 0°.

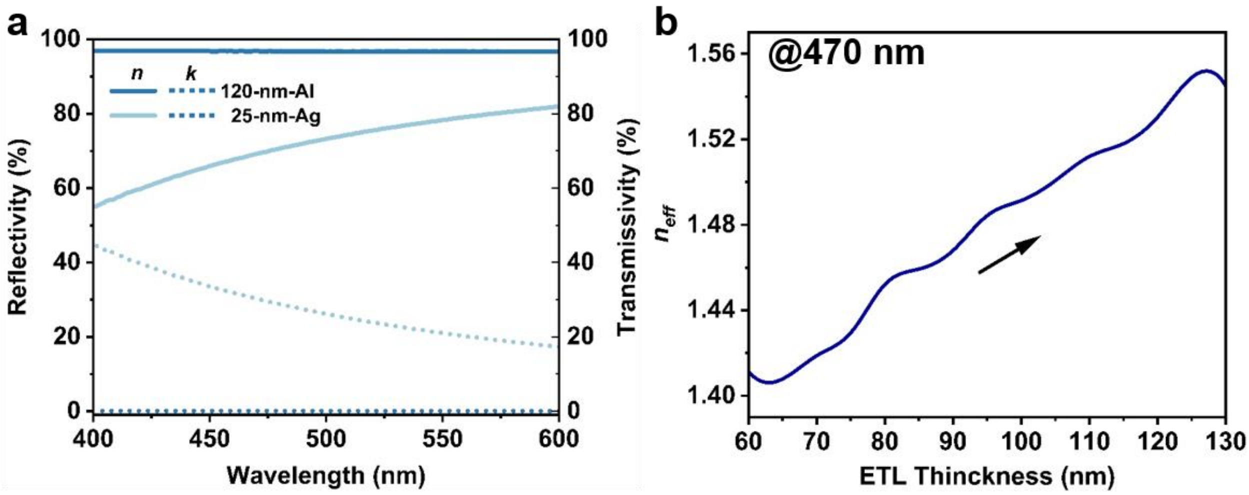

Supplementary Figure3. Design of F-P cavity. (a) Measured transmission (T) and reflectivity (R) of two metal electrodes. 125-nm-Al and 25-nm-Ag correspond to dark and light blue, respectively. (b) Simulated effective refractive index (n_{eff}) of transverse-electric (TE) mode with the variation of electron transport layer (ETL) thickness.

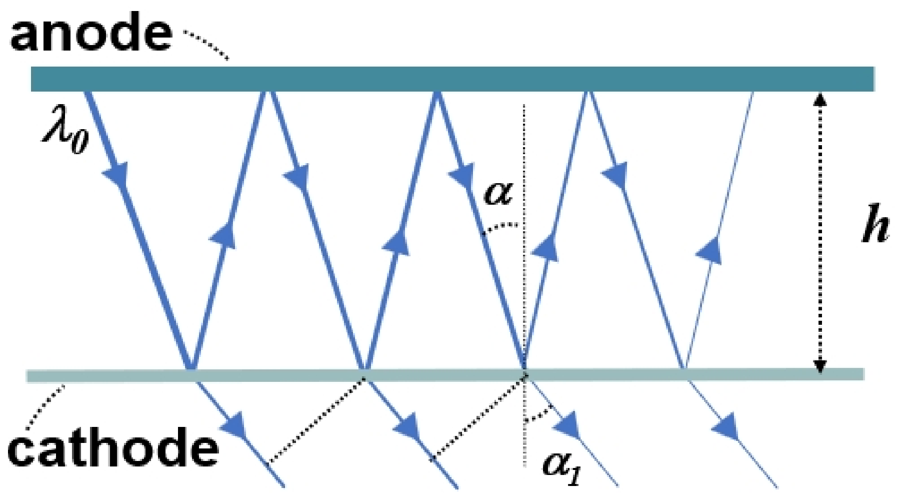

Supplementary Figure 4. The principle of waveguide light emission for F-P nanocavity. Here, α is the incident angle of the photons on the metal film, h the cavity length or organic waveguide (WG) thickness, and λ_0 is the wavelength.

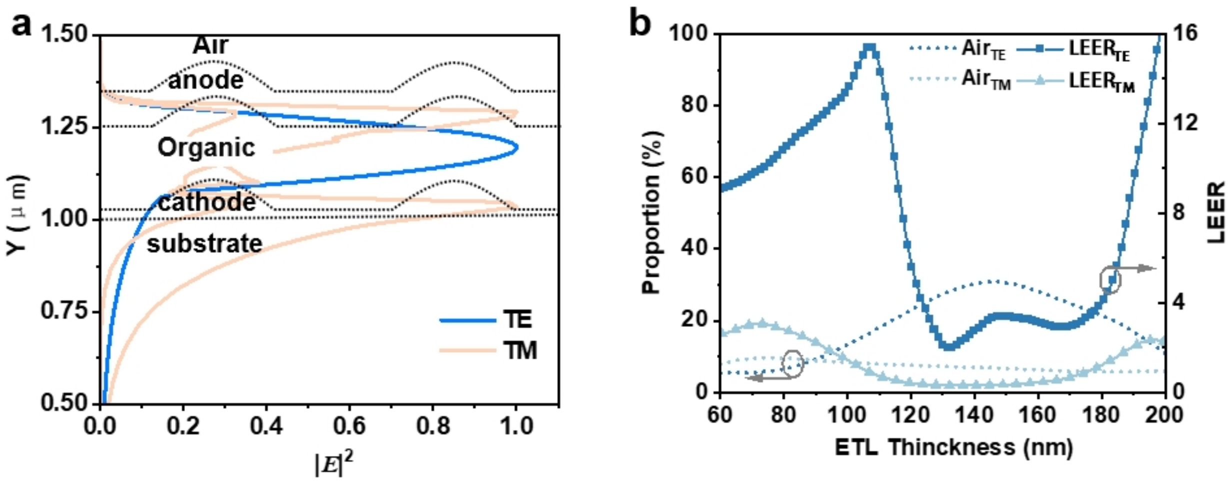

Supplementary Figure 5. Optimization of corrugated OLED. (a) the electric field distribution of the transverse-electric (TE) and transverse-magnetic (TM) modes, based on 107-nm-thick electron transport layer (ETL) and 300-nm-period grating. (b)The air mode (dashed line) distribution and light extraction efficiency enhancement (LEER, solid line). TE polarization is represented by the color dark blue, while TM polarization is represented by the color light blue.

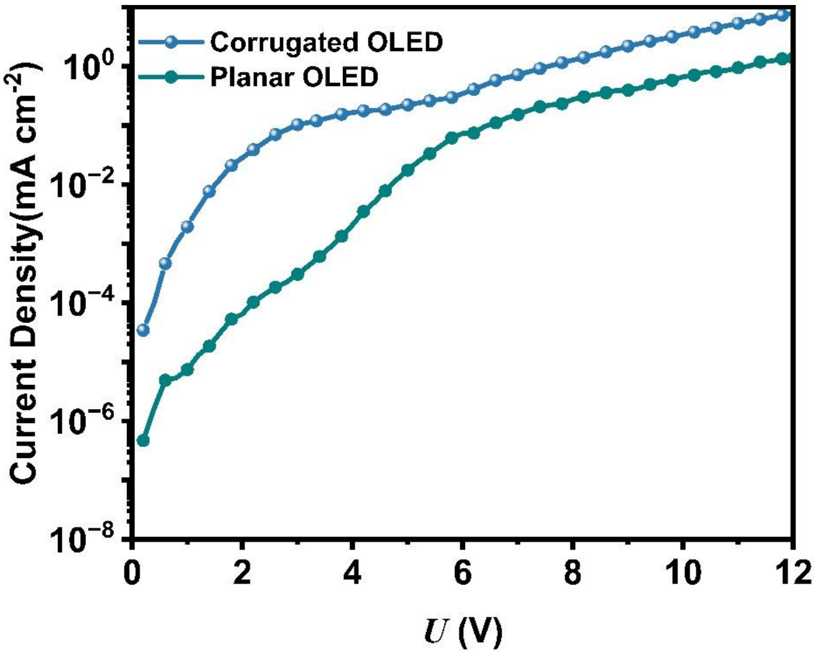

Supplementary Figure 6. Device performance. Current density-Voltage curves for corrugated (blue) and planar OLED (green).

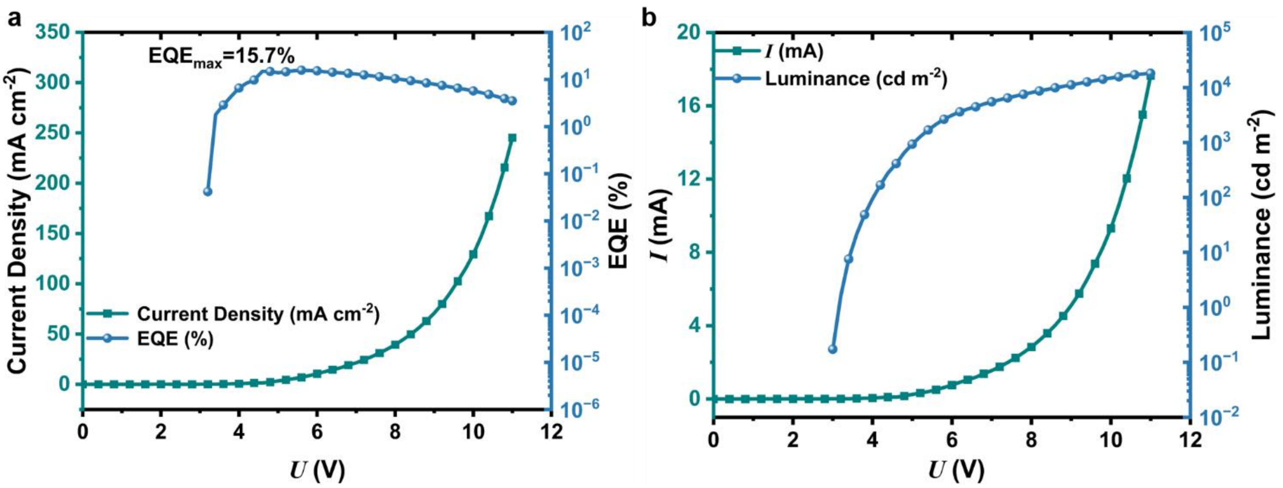

Supplementary Figure 7. Characterization of conventional planar ITO-based OLED with the same FIrpic-based EML. (a) Current density (green)- external quantum efficiency (EQE, blue)-Voltage curves, and (b) Current (*I*, green)-Luminance (blue)-Voltage curves for ITO-based OLEDs. The device structure is ITO/HAT-CN (10 nm)/TAPC (40 nm)/TCTA (5 nm)/TCTA:FIrpic (8%, 15 nm)/mCP:FIrpic (8%, 15 nm)/TmPyPb (40 nm)/LiF (0.8 nm)/Al (120 nm).

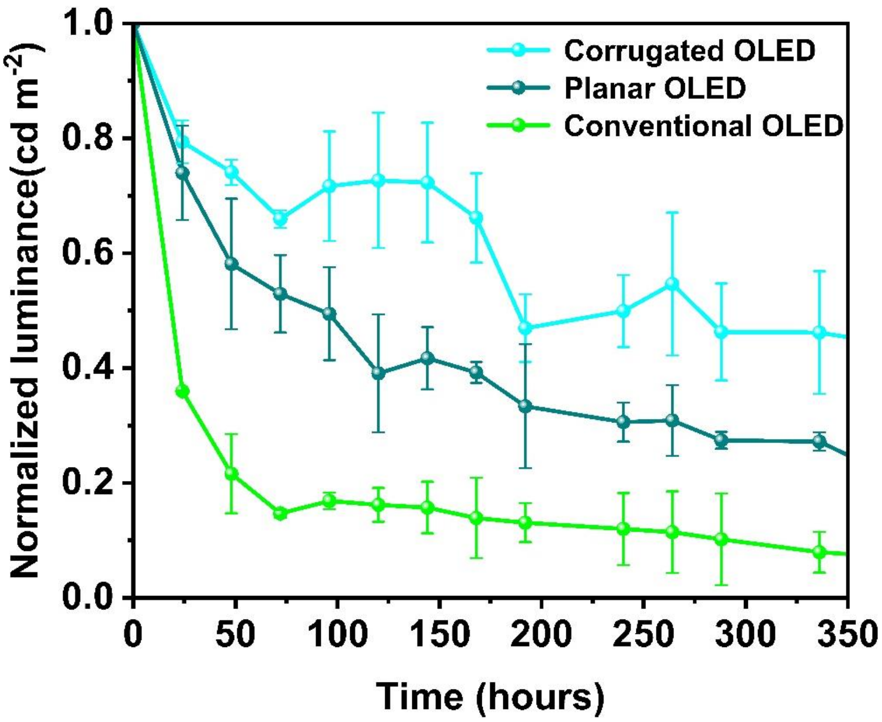

Supplementary Figure 8. Stability operation for unencapsulated OLED stored in nitrogen conditions. Corrugated OLED is represented by the color light blue, planar OLED is represented by the color dark blue, and conventional OLED is represented by green. The error bar is defined as the error generated by testing three devices.

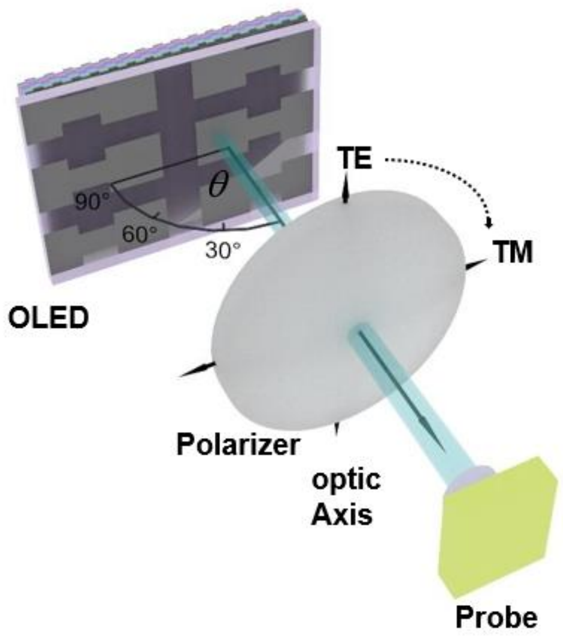

Supplementary Figure 9. **Polarization characterization.** Angular resolved emission spectrum measurement system.

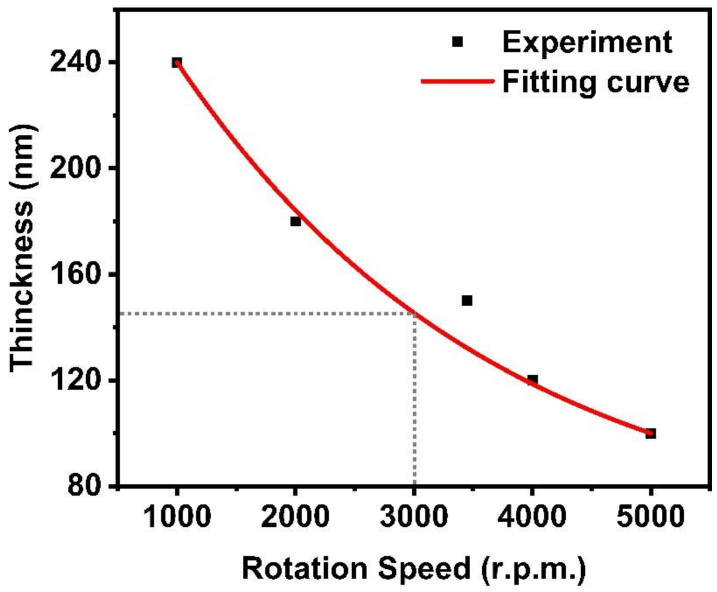

Supplementary Figure 10. The summary of relationship between rotation speed and photoresist thickness. The solid line is the fitting of experimental data (dashed line).

Supplementary Methods

Angle-resolved emission spectroscopy is used to characterize Air mode dispersion under TE and TM polarization. On the one hand, the one-dimensional grating introduces grating momentum only in the horizontal direction, corresponding to the measurement of the polar angle. On the other hand, the polarization emission in the normal direction (with polar angle and azimuth angle equal to 0) was obtained through optimization strategies. As a result, by measuring only the polar angle, we can easily obtain an effective mode dispersion spectrum. The TE light is filtered through the polarizer and the TM light is filtered out by making the fast axis of the polarizer parallel to the grating line of the 1-D grating structure. The TE light is filtered through the polarizer and the TM light is filtered out by making the fast axis of the polarizer parallel to the grating line of the 1-D grating structure. Similarly, the measurement of TM light can be completed by rotating 90° at this position. The probe is connected to a high-precision spectrometer and can be used to detect spectra at specific angles. The angle adjustment can be achieved by changing the angle θ between the device and the probe. Therefore, the cooperative measurement of angle, polarization and wavelength can be realized through this system.

To produce patterned photoresist films on the glass substrate, laser interference lithography was employed. The 145-nm-thick ultrathin positive photoresist films were spin-coated onto the cleaned substrate evenly (3000 r.p.m.) and the thickness can be tuned by rotation speed (Supplementary Fig. 10). We should note that the laser power corresponds to the etching depth. The photoresist will remain in the groove after development, if the photoresist film is too thick, which greatly affects the effective refractive index of the grating.

Supplementary Notes

Supplementary Note 1-Expression of interference equation as a function of grating period with the target resonating wavelength and recording angle

The fabrication of the period is given by the interference equation, which governs the coupling of wavelength λ and angle β to adjust the period grating Λ:

$$\Lambda=\frac{\lambda}{2}\frac{1}{sin\beta} \tag{1}$$

The coated substrates were exposed to the 343 nm laser with a dose of ~80 mJ cm^{-2} and further developed with a diluted developer. The photoresists in the groove were removed mostly and the ridge were well retained under this condition. A large aperture was used to obtain a larger exposure area, corresponding to a larger laser power. Then excess photoresists in the grating groove were etched O_2 plasma process.

Supplementary Note 2-Expression of reflection phase shift as a function of refractive index and incident angle

The reflectivity of the Al electrode increases with increasing wavelength at a thickness of 120 nm, and always above 97% (Supplementary Fig. 3a). By contrast, the reflectivity of the Ag(25 nm) electrode decreased slightly, but remained above 70%. The high reflectivity contributes to the formation of F-P microcavity by two parallel electrodes, which were tuned coordinately by refractive index and cavity length, corresponding to the ETL thickness (Supplementary Fig. 3b). Increasing the thickness of ETL can obtain a greater effective refractive index. We can suppress the air mode emission from the planar OLED by breaking the phase matching. The reflection phase shift in the phase matching condition can be calculated by the following formula, and is sensitive to polarization:

$$\psi_{\mathrm{TE}}=arctan(\frac{-2Bncos\alpha}{n^2cos\alpha-A^2-B^2}) \tag{2}$$

$$\psi_{\mathrm{TM}}=arctan(\frac{n^2}{n_{\mathrm{m}}^2}*\frac{-2Bncos\alpha}{n^2cos\alpha-A^2-B^2}) \tag{3}$$

Where， n,n_{m} is the refractive index of dielectric materials, the real part of metal refractive index, respectively. The parameters A and B are given by：

$$\mathrm{A}=\frac{1}{\sqrt{2}}\sqrt{\sqrt{\left(n_{\mathrm{m}}^2\text{-}k_{\mathrm{m}}^2\text{-}sin\alpha^2\right)+4n_{\mathrm{m}}^2k_{\mathrm{m}}^2}+\left(n_{\mathrm{m}}^2\text{-}k_{\mathrm{m}}^2\text{-}sin\alpha^2\right)} \quad (4)$$

$$\mathrm{B}=\frac{1}{\sqrt{2}}\sqrt{\sqrt{\left(n_{\mathrm{m}}^2\text{-}k_{\mathrm{m}}^2\text{-}sin\alpha^2\right)+4n_{\mathrm{m}}^2k_{\mathrm{m}}^2}\text{-}\left(n_{\mathrm{m}}^2\text{-}k_{\mathrm{m}}^2\text{-}sin\alpha^2\right)} \quad (5)$$

Here the imaginary part of metal refractive index is k_{m}.

Supplementary Table

Supplementary Table 1. Summary performance parameters of linearly polarized electroluminescent devices based on different modulation strategies.

Ref.	Approach	Wavelength (nm)	FWHM (nm)	ER	Device[a]	Polarization
1	Molecular Alignment	447	110	6	OLED	TE/TM
2		575	30	7		
3		520	19	4.8	Perovskite LED	
4		510	70	-10	OLED	TM/TE
5		440	70	-15		
6		493	80	-22		
7		570	210	-14		
8		450	110	-7.8		
9		460	75	-11		
10		520	46	-16.9	GaN LED	
11		540	90	22	OLED	TE/TM
12		511	60	10.8		
13		450	110	13.		
14		630	130	3.8		
15		550	200	5.4		
16	External Nanostructure	520	85	13.9		
17		520	70	16		
18		520	110	-14.6		TM/TE
19		560	150	-17.8		
20		520	65	-20		
21		440	19	-14.1	GaN LED	
22		550	80	-8.5	InGaN LED	
23	Embedded Nanostructure	520	20	11.1	OLED	TE/TM
24		530	100	4.8		
This work		470	28	15.8		

[a] ER is defined as ER=$10log\frac{I_{TE}}{I_{TM}}$; the negative values represent the ER obtained from $10log\frac{I_{TM}}{I_{TE}}$.

Supplementary References

1. Clauswitz, K. U. W., Geffarth, F., Greiner, A., Lüssem, G. & Wendorff, J. H. Polarized electroluminescence from liquid crystalline polymers. *Synth. Met*. **111-112**, 169-171 (2000).
2. Ding, R. et al. Intrinsic Polarization and Tunable Color of Electroluminescence from Organic Single Crystal-based Light Emitting Devices. *Sci. Rep.* **5**, 12445 (2015).
3. Zhang, J. et al. Strong Linearly Polarized Photoluminescence and Electroluminescence from Halide Perovskite/Azobenzene Dye Composite Film for Display Applications. *Adv. Opt. Mater.* **8**, 1901824 (2020).
4. Contoret, A. E. A. et al. Polarized Electroluminescence from an Anisotropic Nematic Network on a Non-contact Photoalignment Layer. *Adv. Mater*. **12**, 971-974 (2000).
5. Misaki, M. et al. Highly efficient polarized polymer light-emitting diodes utilizing oriented films of β-phase poly(9,9-dioctylfluorene). *Appl. Phys. Lett.* **93**, 023304 (2008).
6. An, M. H. et al. Highly polarized emission from organic single-crystal light-emitting devices with a polarization ratio of 176. *Optica.* **9**, 121-129 (2022).
7. Chen, A. C. A. et al. Organic Polarized Light-Emitting Diodes via Förster Energy Transfer Using Monodisperse Conjugated Oligomers†. *Adv. Mater*. **16**, 783-788 (2004).
8. Lüssem, G. et al. Polarized electroluminescence of light emitting liquid crystalline polymers. *Liq. Cryst*. **21**, 903-907 (1996).
9. Sainova, D. et al. Photoaddressable Alignment Layers for Fluorescent Polymers in Polarized Electroluminescence Devices. *Adv. Funct. Mater*. **12**, 49-57 (2002).
10. Brinkley, S. E. et al. Polarized spontaneous emission from blue-green m-plane GaN-based light emitting diodes. *Appl. Phys. Lett.* **98**, 011110 (2011).
11. Choi, G. J. et al. Polarized light-emitting diodes based on patterned MoS2 nanosheet hole transport layer. *Adv. Mater*. **29**, 1702598 (2017).
12. Jandke, M., Strohriegl, P., Gmeiner, J., Brütting, W. & Schwoerer, M. Polarized electroluminescence from rubbing-aligned poly(p-phenylenevinylene). *Synth. Met.* **111-112**, 177-180 (2000).
13. Miteva, T. et al. Polarized electroluminescence from highly aligned liquid crystalline polymers. *Synth. Met.* **111-112**, 173-176 (2000).
14. Dyreklev, P. et al. Polarized electroluminescence from an oriented substituted polythiophene in a light emitting diode. *Adv. Mater*. **7**, 43-45 (1995).

15. Cimrová, V., Remmers, M., Neher, D. & Wegner, G. Polarized light emission from LEDs prepared by the Langmuir-Blodgett technique. *Adv. Mater*. **8**, 146-149 (1996).
16. Park, B., Park, C. H., Kim, M. & Han, M. Y. Polarized organic light-emitting device on a flexible giant birefringent optical reflecting polarizer substrate. *Opt. Express*. **17**, 10136-10143 (2009).
17. Park, B., Huh, Y. H. & Jeon, H. G. Polarized electroluminescence from organic light-emitting devices using photon recycling. *Opt. Express*. **18**, 19824-19830 (2010).
18. Lin, M. Y. et al. White Organic Light-Emitting Diode With Linearly Polarized Emission. *IEEE Photon. Technol. Lett.* **25**, 1321-1323 (2013).
19. Zhou, L. et al. Tailored polarization conversion and light-energy recycling for highly linearly polarized white organic light-emitting diodes. *Laser Photonics Rev*. **14**, 1900341 (2020).
20. Zhou, L. et al. Highly linearly polarized light emission from flexible organic light-emitting devices capitalized on integrated ultrathin metal-dielectric nanograting. *Opt. Express*. **28**, 13826-13836 (2020).
21. Huang, J. P. et al. Linearly polarized light emission from GaN micro-LEDs for 3D display. *Appl. Phys. Lett*. **122**, 111107 (2023).
22. Zhang, L., Teng, J. H., Chua, S. J. & Eugene, A. F. Linearly polarized light emission from InGaN light emitting diode with subwavelength metallic nanograting. *Appl. Phys. Lett.* **95**, 261110 (2009).
23. Fu, X. Y. et al. Directional Polarized Light Emission from Thin-Film Light-Emitting Diodes. *Adv. Mater*. **33**, 2006801 (2021).
24. Lin, B. Y. et al. Effects of electron transport layer thickness on light extraction in corrugated OLEDs. *Opt. Express*. **30**, 18066-18078 (2022).
