# Supplementary material for: Dual-color emissive OLED with orthogonal polarization modes
Source: Nat Commun. 2024 Feb 13;15:1331. doi: 10.1038/s41467-024-45311-1 (PMC10864411; doi:10.1038/s41467-024-45311-1)
Supplement: Supplementary file 3 — Description of Additional Supplementary Files [file 41467_2024_45311_MOESM3_ESM.pdf]

Description of Additional Supplementary Files

Supplementary Software 1. Simulated code and diagram of results.

Supplementary Movie 1. The diagram of color conversion in LP-OLED.
