# Supplementary material for: Dual-color emissive OLED with orthogonal polarization modes
Source: Nat Commun. 2024 Feb 13;15:1331. doi: 10.1038/s41467-024-45311-1 (PMC10864411; doi:10.1038/s41467-024-45311-1)

For corrugated OLEDs, model in FDTD using the following code:

select("granting");

delete;

select("mesh_cathode");

delete;

select("mesh_dipole");

delete;

addstructuregroup;

set("name","granting");

set("x",0);

set("y",0);

set("z",0);

um=1e-6;

nm=1e-9;

f_2=1-f;

addrect;

set("name","glass");

set("x",0);

set("y",0);

set("z",0);

set("x span",k*a);

set("y span",glass_span);

set("z span",c_z);

set("material","SiO2 (Glass) - Palik");

glass_y=getnamed("glass","y");

addtogroup("granting");

z=(k-1)/2;

u=(j-1)/2;

n=a*f/j;

for(i=-z:z){

x_1=a*i;

x_2=a*i+a/2;

for(p=-u:u){

MgF2_y=glass_y+glass_span/2+(MgF2*cos((p/(j-1))*pi))/2;

addrect;

set("name","MgF2");

set("x",x_1+p*n);

set("y",MgF2_y);

set("z",0);

set("x span",n);

set("y span",MgF2*cos((p/(j-1))*pi));

set("z span",c_z);

set("material","MgF2");

addtogroup("granting");

cathode_y_1=MgF2_y+(MgF2*cos((p/(j-1))*pi))/2+cathode/2;

addrect;

set("name","cathode_1");

set("x",x_1+p*n);

set("y",cathode_y_1);

set("z",0);

set("x span",n);

set("y span",cathode);

set("z span",c_z);

set("material","Ag (Silver) - Palik (0-2um)");

addtogroup("granting");

cathode_2_y_1=cathode_y_1+cathode/2+cathode_2/2;

addrect;

set("name","cathode_1");

set("x",x_1+p*n);

set("y",cathode_2_y_1);

set("z",0);

set("x span",n);

set("y span",cathode_2);

set("z span",c_z);

set("material"," Ag (Silver) - Palik (0-2um)");

addtogroup("granting");

LiF_y_1=cathode_2_y_1+LiF/2+cathode_2/2;

addrect;

set("name","LiF_1");

set("x",x_1+p*n);

set("y",LiF_y_1);

set("z",0);

set("x span",n);

set("y span",LiF);

set("z span",c_z);

set("material","LiF");

addtogroup("granting");

TmPyPb_y_1=LiF_y_1+LiF/2+TmPyPb/2;

addrect;

set("name","TmPyPb_1");

set("x",x_1+p*n);

set("y",TmPyPb_y_1);

set("z",0);

set("x span",n);

set("y span",TmPyPb);

set("z span",c_z);

set("material","TmPyPb");

addtogroup("granting");

mCP_y_1=TmPyPb_y_1+mCP/2+TmPyPb/2;

addrect;

set("name","mCP_1");

set("x",x_1+p*n);

set("y",mCP_y_1);

set("z",0);

set("x span",n);

set("y span",mCP);

set("z span",c_z);

set("material","mCP");

addtogroup("granting");

TCTA_y_1=mCP_y_1+mCP/2+TCTA/2;

addrect;

set("name","TCTA_1");

set("x",x_1+p*n);

set("y",TCTA_y_1);

set("z",0);

set("x span",n);

set("y span",TCTA);

set("z span",c_z);

set("material","TCTA1");

addtogroup("granting");

TAPC_y_1=TCTA_y_1+TAPC/2+TCTA/2;

addrect;

set("name","TAPC_1");

set("x",x_1+p*n);

set("y",TAPC_y_1);

set("z",0);

set("x span",n);

set("y span",TAPC);

set("z span",c_z);

set("material","TAPC");

addtogroup("granting");

HATCN_y_1=TAPC_y_1+TAPC/2+HATCN/2;

addrect;

set("name","HATCN_1");

set("x",x_1+p*n);

set("y",HATCN_y_1);

set("z",0);

set("x span",n);

set("y span",HATCN);

set("z span",c_z);

set("material","HATCN");

addtogroup("granting");

anode_y_1=HATCN_y_1+HATCN/2+anode/2;#The center y of ITO

addrect;

set("name","anode_1");

set("x",x_1+p*n);

set("y",anode_y_1);

set("z",0);

set("x span",n);

set("y span",anode);

set("z span",c_z);

set("material","Al (Aluminium) - Palik");

addtogroup("granting");

anode_2_y_1=anode_y_1+anode/2+anode_2/2;#The center y of ITO

addrect;

set("name","anode_1");

set("x",x_1+p*n);

set("y",anode_2_y_1);

set("z",0);

set("x span",n);

set("y span",anode_2);

set("z span",c_z);

set("material","Al (Aluminium) - Palik");

addtogroup("granting");

}

cathode_y_2=glass_y+glass_span/2+cathode/2;

addrect;

set("name","cathode_2");

set("x",x_2);

set("y",cathode_y_2);

set("z",0);

set("x span",a*f_2);

set("y span",cathode);

set("z span",c_z);

set("material","Ag (Silver) - Palik (0-2um)");

addtogroup("granting");

cathode_2_y_2=cathode_y_2+cathode/2+cathode_2/2;

addrect;

set("name","cathode_2");

set("x",x_2);

set("y",cathode_2_y_2);

set("z",0);

set("x span",a*f_2);

set("y span",cathode_2);

set("z span",c_z);

set("material"," Ag (Silver) - Palik (0-2um)");

addtogroup("granting");

LiF_y_2=cathode_2_y_2+LiF/2+cathode_2/2;

addrect;

set("name","LiF_2");

set("x",x_2);

set("y",LiF_y_2);

set("z",0);

set("x span",a*f_2);

set("y span",LiF);

set("z span",c_z);

set("material","LiF");

addtogroup("granting");

TmPyPb_y_2=LiF_y_2+LiF/2+TmPyPb/2;

addrect;

set("name","TmPyPb_2");

set("x",x_2);

set("y",TmPyPb_y_2);

set("z",0);

set("x span",a*f_2);

set("y span",TmPyPb);

set("z span",c_z);

set("material","TmPyPb");

addtogroup("granting");

mCP_y_2=TmPyPb_y_2+TmPyPb/2+mCP/2;

addrect;

set("name","mCP_2");

set("x",x_2);

set("y",mCP_y_2);

set("z",0);

set("x span",a*f_2);

set("y span",mCP);

set("z span",c_z);

set("material","mCP");

addtogroup("granting");

TCTA_y_2=mCP_y_2+mCP/2+TCTA/2;

addrect;

set("name","TCTA_2");

set("x",x_2);

set("y",TCTA_y_2);

set("z",0);

set("x span",a*f_2);

set("y span",TCTA);

set("z span",c_z);

set("material","TCTA1");

addtogroup("granting");

TAPC_y_2=TCTA_y_2+TAPC/2+TCTA/2;

addrect;

set("name","TAPC_2");

set("x",x_2);

set("y",TAPC_y_2);

set("z",0);

set("x span",a*f_2);

set("y span",TAPC);

set("z span",c_z);

set("material","TAPC");

addtogroup("granting");

HATCN_y_2=TAPC_y_2+TAPC/2+HATCN/2;

addrect;

set("name","HATCN_2");

set("x",x_2);

set("y",HATCN_y_2);

set("z",0);

set("x span",a*f_2);

set("y span",HATCN);

set("z span",c_z);

set("material","HATCN");

addtogroup("granting");

anode_y_2=HATCN_y_2+HATCN/2+anode/2;

addrect;

set("name","anode_2");

set("x",x_2);

set("y",anode_y_2);

set("z",0);

set("x span",a*f_2);

set("y span",anode);

set("z span",c_z);

set("material","Al (Aluminium) - Palik");

addtogroup("granting");

anode_2_y_2=anode_y_2+anode/2+anode_2/2;

addrect;

set("name","anode_2");

set("x",x_2);

set("y",anode_2_y_2);

set("z",0);

set("x span",a*f_2);

set("y span",anode_2);

set("z span",c_z);

set("material","Al (Aluminium) - Palik");

addtogroup("granting");

}

For planar OLEDs, model in FDTD using the following code:

select("granting");

delete;

select("mesh_cathode");

delete;

select("mesh_dipole");

delete;

addstructuregroup;

set("name","granting");

set("x",0);

set("y",0);

set("z",0);

um=1e-6;

nm=1e-9;

f_2=1-f;

addrect;

set("name","glass");

set("x",0);

set("y",0);

set("z",0);

set("x span",x2);

set("y span",glass_span);

set("z span",c_z);

set("material","SiO2 (Glass) - Palik");

glass_y=getnamed("glass","y");

addtogroup("granting");

addrect;

set("name","cathode_2");

set("x",0);

set("y",cathode_y_2);

set("z",0);

set("x span",x2);

set("y span",cathode);

set("z span",c_z);

set("material","Ag (Silver) - Palik (0-2um)");

addtogroup("granting");

cathode_2_y_2=cathode_y_2+cathode/2+cathode_2/2;

addrect;

set("name","cathode_2");

set("x",0);

set("y",cathode_2_y_2);

set("z",0);

set("x span",x2);

set("y span",cathode_2);

set("z span",c_z);

set("material"," Ag (Silver) - Palik (0-2um)");

addtogroup("granting");

LiF_y_2=cathode_2_y_2+LiF/2+cathode_2/2;

addrect;

set("name","LiF_2");

set("x",0);

set("y",LiF_y_2);

set("z",0);

set("x span",x2);

set("y span",LiF);

set("z span",c_z);

set("material","LiF");

addtogroup("granting");

TmPyPb_y_2=LiF_y_2+LiF/2+TmPyPb/2;

addrect;

set("name","TmPyPb_2");

set("x",0);

set("y",TmPyPb_y_2);

set("z",0);

set("x span",x2);

set("y span",TmPyPb);

set("z span",c_z);

set("material","TmPyPb");

addtogroup("granting");

mCP_y_2=TmPyPb_y_2+TmPyPb/2+mCP/2;

addrect;

set("name","mCP_2");

set("x",0);

set("y",mCP_y_2);

set("z",0);

set("x span",x2);

set("y span",mCP);

set("z span",c_z);

set("material","mCP");

addtogroup("granting");

TCTA_y_2=mCP_y_2+mCP/2+TCTA/2;

addrect;

set("name","TCTA_2");

set("x",0);

set("y",TCTA_y_2);

set("z",0);

set("x span",x2);

set("y span",TCTA);

set("z span",c_z);

set("material","TCTA1");

addtogroup("granting");

TAPC_y_2=TCTA_y_2+TAPC/2+TCTA/2;

addrect;

set("name","TAPC_2");

set("x",0);

set("y",TAPC_y_2);

set("z",0);

set("x span",x2);

set("y span",TAPC);

set("z span",c_z);

set("material","TAPC");

addtogroup("granting");

HATCN_y_2=TAPC_y_2+TAPC/2+HATCN/2;

addrect;

set("name","HATCN_2");

set("x",0);

set("y",HATCN_y_2);

set("z",0);

set("x span",x2);

set("y span",HATCN);

set("z span",c_z);

set("material","HATCN");

addtogroup("granting");

anode_y_2=HATCN_y_2+HATCN/2+anode/2;

addrect;

set("name","anode_2");

set("x",0);

set("y",anode_y_2);

set("z",0);

set("x span",x2);

set("y span",anode);

set("z span",c_z);

set("material","Al (Aluminium) - Palik");

addtogroup("granting");

We have included additional simulation details in the theoretical modeling section of the main text. All parameters utilized in the code, such as film thickness and period, have been explicitly stated. For instance, the following layer sequence was employed: Ag (25 nm)/LiF (0.8 nm)/TmPyPb (107 nm)/mCP:FIrpic (8%, 15 nm)/TCTA:FIrpic (8%, 15 nm)/TCTA (5 nm)/TAPC (40 nm)/HAT-CN (10 nm)/Al (120 nm). Based on the above structure, the far-field emission of corrugated OLEDs is simulated. For example, TE emission of corrugated OLED:


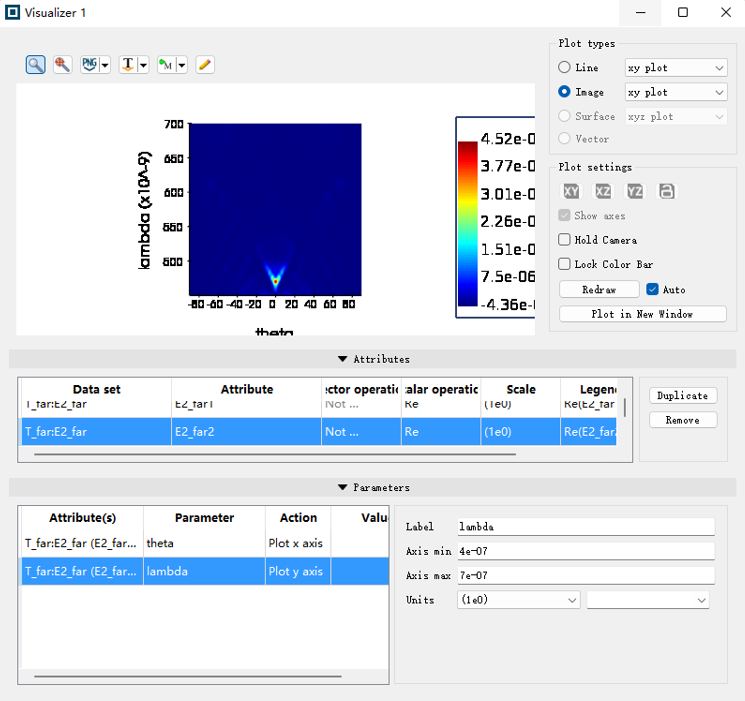


The TM emission of corrugated OLED:
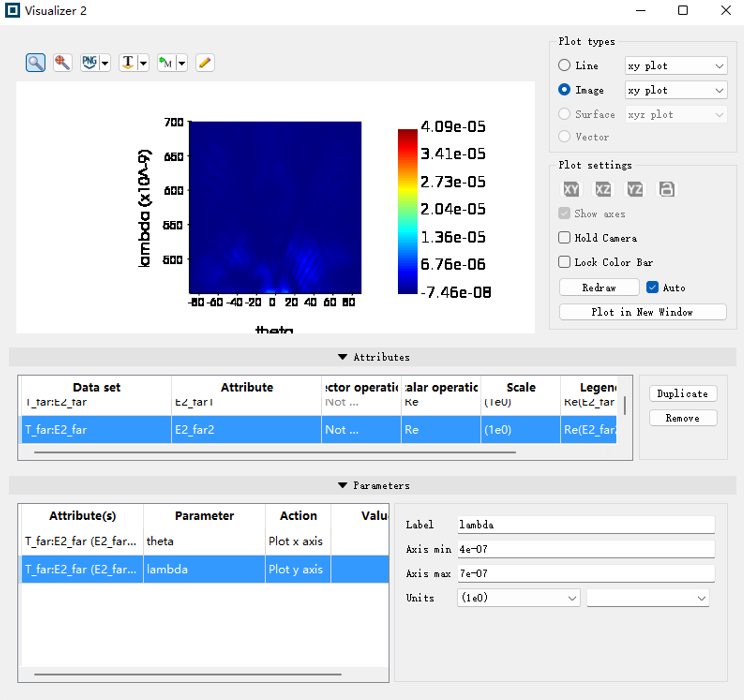

Supplement: Supplementary file 4 — Supplementary Software 1 [file 41467_2024_45311_MOESM4_ESM.zip › Supplementary Software 1/Supplementary Code.docx]
