# Supplementary material for: Dual-color emissive OLED with orthogonal polarization modes
Source: Nat Commun. 2024 Feb 13;15:1331. doi: 10.1038/s41467-024-45311-1 (PMC10864411; doi:10.1038/s41467-024-45311-1)
Supplement: Supplementary file 6 — Reporting Summary [file 41467_2024_45311_MOESM6_ESM.pdf]

nature portfolio

Corresponding author(s): NCOMMS-23-36627A
Tianrui Zhai and Ningning Liang

Last updated by author(s): Dec 12, 2023

Reporting Summary

Nature Portfolio wishes to improve the reproducibility of the work that we publish. This form provides structure for consistency and transparency in reporting. For further information on Nature Portfolio policies, see our Editorial Policies and the Editorial Policy Checklist.

Statistics

For all statistical analyses, confirm that the following items are present in the figure legend, table legend, main text, or Methods section.

n/a	Confirmed	
☐	☒	The exact sample size (*n*) for each experimental group/condition, given as a discrete number and unit of measurement
☐	☒	A statement on whether measurements were taken from distinct samples or whether the same sample was measured repeatedly
☐	☒	The statistical test(s) used AND whether they are one- or two-sided<br>*Only common tests should be described solely by name; describe more complex techniques in the Methods section.*
☐	☒	A description of all covariates tested
☒	☐	A description of any assumptions or corrections, such as tests of normality and adjustment for multiple comparisons
☐	☒	A full description of the statistical parameters including central tendency (e.g. means) or other basic estimates (e.g. regression coefficient) AND variation (e.g. standard deviation) or associated estimates of uncertainty (e.g. confidence intervals)
☒	☐	For null hypothesis testing, the test statistic (e.g. *F*, *t*, *r*) with confidence intervals, effect sizes, degrees of freedom and *P* value noted<br>*Give P values as exact values whenever suitable.*
☒	☐	For Bayesian analysis, information on the choice of priors and Markov chain Monte Carlo settings
☒	☐	For hierarchical and complex designs, identification of the appropriate level for tests and full reporting of outcomes
☒	☐	Estimates of effect sizes (e.g. Cohen's *d*, Pearson's *r*), indicating how they were calculated

Our web collection on statistics for biologists contains articles on many of the points above.

Software and code

Policy information about availability of computer code

Data collection	The calculation software used is MATLAB and FDTD. All software can be downloaded and detailed explanations are provided in the article.
Data analysis	All data used in our manuscript is publicly available and provided in the supporting information file. This information is also explicitly mentioned in the data and code availability section of the manuscript.

For manuscripts utilizing custom algorithms or software that are central to the research but not yet described in published literature, software must be made available to editors and reviewers. We strongly encourage code deposition in a community repository (e.g. GitHub). See the Nature Portfolio guidelines for submitting code & software for further information.

Data

Policy information about availability of data

All manuscripts must include a data availability statement. This statement should provide the following information, where applicable:

- Accession codes, unique identifiers, or web links for publicly available datasets
- A description of any restrictions on data availability
- For clinical datasets or third party data, please ensure that the statement adheres to our policy

The authors declare that the main data supporting the findings of this study are available within the article and its Supplementary Information files. The source data underlying Figs. 1b, 2a, 2d, 2g, 3c, 3e, 3f, 3g, 4f, s1b, s2a, s4a,s4b, s5-7,s9 are provided in the Source Data files with this paper or extra data are available from the corresponding author on request. All figures and data are original and there is no third-party data available.

Research involving human participants, their data, or biological material

Policy information about studies with human participants or human data. See also policy information about sex, gender (identity/presentation), and sexual orientation and race, ethnicity and racism.

Reporting on sex and gender	not applicable
Reporting on race, ethnicity, or other socially relevant groupings	not applicable
Population characteristics	not applicable
Recruitment	not applicable
Ethics oversight	not applicable

Note that full information on the approval of the study protocol must also be provided in the manuscript.

Field-specific reporting

Please select the one below that is the best fit for your research. If you are not sure, read the appropriate sections before making your selection.

☐ Life sciences ☐ Behavioural & social sciences ☒ Ecological, evolutionary & environmental sciences

For a reference copy of the document with all sections, see nature.com/documents/nr-reporting-summary-flat.pdf

Ecological, evolutionary & environmental sciences study design

All studies must disclose on these points even when the disclosure is negative.

Study description	All data was independently tested by the research group. Previously, researchers have proposed various methods to achieve linearly polarized OLED, but few have studied dual-color orthogonal polarized OLED. This study aims to achieve dual-color orthogonal linearly polarized OLED with high efficiency emission.
Research sample	All samples were self-made. The preparation method is described in the Methods section of the article.
Sampling strategy	We conducted multiple measurements on all samples to ensure the accuracy of the data.
Data collection	The equipment used for data collection, such as spectroscopy, SEM, and AFM, has been presented in the Characterization of OLED section of the article. The testers are all authors of the article.
Timing and spatial scale	not applicable
Data exclusions	No data excluded
Reproducibility	All OLED samples were prepared using vacuum thermal evaporation method, which has high reproducibility. For the preparation of gratings, the period has extremely high repeatability, and the variation of surface roughness has little effect on the experimental results. We prepared multiple samples under the same conditions to ensure data reproducibility.
Randomization	not applicable
Blinding	not applicable

Did the study involve field work? ☐ Yes ☒ No

Reporting for specific materials, systems and methods

We require information from authors about some types of materials, experimental systems and methods used in many studies. Here, indicate whether each material, system or method listed is relevant to your study. If you are not sure if a list item applies to your research, read the appropriate section before selecting a response.

Materials & experimental systems

n/a	Involved in the study
☒	☐ Antibodies
☒	☐ Eukaryotic cell lines
☒	☐ Palaeontology and archaeology
☒	☐ Animals and other organisms
☒	☐ Clinical data
☒	☐ Dual use research of concern
☒	☐ Plants

Methods

n/a	Involved in the study
☒	☐ ChIP-seq
☒	☐ Flow cytometry
☒	☐ MRI-based neuroimaging

Plants

Seed stocks	not applicable
Novel plant genotypes	not applicable
Authentication	not applicable
